# Trends in the Incidence of Vulvar and Vaginal Cancers With Different Histology by Race, Age, and Region in the United States (2001–2018)

**DOI:** 10.3389/ijph.2022.1605021

**Published:** 2022-08-29

**Authors:** Wei-Li Zhou, Yang-Yang Yue

**Affiliations:** ^1^ Department of General Surgery, ShengJing Hospital of China Medical University, Shenyang, China; ^2^ Department of Health Management, ShengJing Hospital of China Medical University, Shenyang, China

**Keywords:** trends, incidence rate, vaginal cancer, squamous cell carcinoma, adenocarcinoma, vulvar cancer

## Abstract

**Objectives:** The race, age, and region-stratified incidence of vulvar (VUC) and vaginal (VAC) cancers with different histology were unclear.

**Methods:** Data was retrieved from the United States Cancer Statistics database. Average annual percent change (AAPC) and incidence rate ratio (IRR) were calculated.

**Results:** Overall, VUC incidence increased from 18.3 (per 1,000,000 woman-years) to 19.6, but VAC incidence decreased from 5.6 to 4.4. VUC squamous cell carcinoma (SCC) incidence increased (AAPC, 0.96; 95% CI, 0.66–1.25), VUC adenocarcinoma (ADE) incidence stabilized (AAPC, −0.24; 95% CI, −1.44 to 0.98), and VUC other malignancies (OM) incidence decreased (AAPC, −1.31; 95% CI, −2.58 to −0.02). While VAC incidence decreased for any histology (AAPC, -0.63; 95% CI, −1.03 to −0.22; AAPC, −1.60; 95% CI, −2.80 to −0.39; and AAPC, −1.57; 95% CI, −2.24 to −0.89 for SCC, ADE, and OM). Similar trends were observed in most of the stratifications.

**Conclusion:** VUC and VAC incidences varied by histology overall and within stratifications by race, age, and region. The incidence decreased for VUC and VAC with all histologies, except for the increasing VUC SCC incidence.

## Introduction

Vulvar cancer (VUC) and vaginal cancer (VAC) are two rare malignancies in women, accounting for nearly 10% of all gynecologic cancers, with approximately 6000 newly diagnosed cases and 5000 deaths yearly in the United States [1]. Squamous cell carcinoma (SCC, 80%–90%) is the primary histological type of these two carcinomas, followed by adenocarcinoma (ADE, 4%–10%) and other histological types (hereafter referred to as other malignancies, OM) [2–4].

From 1999 to 2015 in the United States, the incidence rate of VUC SCC increased by 1.3% per year, with a significant increase in women aged 50–69. The incidence rate of VAC SCC decreased by 0.3% per year, with a significant decrease in women aged younger than 40 and 70 years or older [5]. However, no studies investigate the incidence rate trends of ADE and OM, especially within different age groups.

It has been reported that 69% and 71% of VUC and VAC patients have an HPV infection, indicating that VUC and VAC are probably highly associated with human papillomavirus (HPV) [6]. Thus, disparities in HPV prevalence and HPV vaccination rate could probably contribute to the disparities in the incidence rate of these two cancers. Furthermore, geographic disparities in vaccination uptake across different United States regions may also result in continuing disparities in VUC and VAC [7, 8]. Thus, investigating the incidence rate of these two cancers within different geographic regions of the United States may provide some clues.

Racial disparities in incidence rates are well documented in several common carcinomas, such as malignant meningioma, hepatocellular carcinoma, uterine corpus carcinoma, prostate carcinoma, and pancreatic carcinoma [9–14]. Referring to the VUC and VAC, although the trends in the incidence rate of SCC have been assessed, the incidence rate trends in ADE and OM have not been investigated, especially within subgroups stratified by race.

To our knowledge, previous studies have not comprehensively taken histological type, age, race, and geographic region into account when evaluating the incidence rate trends of these two cancers. Thus, this study aimed to evaluate the age-adjusted incidence rate trends of VUC and VAC with different histological subtypes overall and stratified by race, age, and geographic regions of the United States.

## Methods

### Study Population

Using the third edition of the International Classification of Diseases for Oncology (ICD-O-3) codes, we identified patients with microscopically confirmed primary VUC (including labium majus [C51.0], labium minus [C51.1], clitoris [C51.2], overlapping lesion of the vulva [C51.8], and vulva NOS [C51.9]) and primary VAC (C52.9) diagnosed between 1 January 2001 and 31 December 2018 from the United States Cancer Statistics (USCS, representing approximately 97.8% of the United States population) database (2020 submission). We included patients aged 20 years or older and excluded patients aged younger than 20 years due to the small case counts in this age group. Patients whose cancer was reported through only a death certificate or autopsy were also included. Patients with a primary tumor *in situ* were excluded. Age was classified into six categories: 20–39, 40–49, 50–59, 60–69, 70–79, and 80 years or older. The race included three groups-whites, blacks, and others (American Indians/Alaska Native, Asian or Pacific Islander, and Unknown race). Cases were classified into three subgroups by histology based on the ICD-O-3 histology codes: SCC (8050-8089), ADE (8140–8389), and OM (8000–8049, 8090–8139, 8390–9992). ADE and OM were merged into a group named non-SCC when evaluating the incidence rate trends within subgroups stratified by race, age, and region, because of cases of less than 16 in some locations. The regions of the United States include the Northeast (Connecticut, Massachusetts, Maine, New Hampshire, New Jersey, New York, Pennsylvania, Rhode Island, Vermont), Midwest (Illinois, Indiana, Iowa, Kansas, Michigan, Minnesota, Missouri, North Dakota, Nebraska, Ohio, South Dakota, and Wisconsin), South (Alabama, Arkansas, District of Columbia, Delaware, Florida, Georgia, Kentucky, Louisiana, Maryland, Mississippi, North Carolina, Oklahoma, South Carolina, Tennessee, Texas, Virginia, and West Virginia), and the West (Alaska, Arizona, California, Colorado, Hawaii, Idaho, Montana, New Mexico, Nevada, Oregon, Utah, Washington, and Wyoming). The ethics approval was waived by the Ethics Committee of the ShengJing Hospital of China Medical University because we use public available deidentified data.

### Statistical Analysis

The age-adjusted incidence rates were calculated using the SEER*Stat software (version 8.3.9) overall and by race, histological subtype, age group, and region. Incidence rates were age-adjusted to the 2000 US standard population and expressed as per 1,000,000 woman-years. Incidence rate ratios (IRR) were estimated, and their 95% confidence intervals (CIs) were calculated using the Tiwari modification method.

Trends in incidence rates were calculated using the National Cancer Institute Joinpoint regression software (version 4.9.0.0). In the Joinpoint software, we calculated annual percent changes (APCs) and their 95% CIs using the Empirical Quantile method [15, 16]. The Joinpoint software chooses the best-fitting linear regression model in a logarithmic scale based on the least-weighted Bayesian Information Criterion to identify years when APCs significantly changed, allowing a maximum of three joinpoints while providing a minimum number of joinpoints necessary to fit the data. Trends were summarized by the average APCs (AAPCs). All statistical tests were two-sided, and a *p*-value of less than 0.05 was considered statistically significant.

## Results

### Age-Adjusted Incidence Rates

We identified 81636 primary VUC cases and 21954 primary VAC cases from the USCS database between 2001 and 2018. Overall, the age-adjusted VUC incidence rate was 19.2 per 1,000,000 woman-years, and the VAC incidence rate was 5.1 per 1,000,000 woman-years. The incidence rates varied considerably by race, with the highest VUC incidence rate among whites (20.3) and the highest VAC incidence rate among blacks (7.6). Moreover, the lowest incidence rates for VUC and VAC were demonstrated in others (10.9 and 4.3, respectively). The incidence rate varied widely by histological type and was highest for SCC (15.1 and 3.4 for VUC and VAC, respectively), followed by OM (3.8 and 1.0) and ADE (0.3 and 0.8) (Table 1).

**TABLE 1:** Age-adjusted incidence rates of vulvar and vaginal cancers by histology and race (cohort study, United States, 2001–2018).

Category	Vulvar cancer			Vaginal cancer		
	No. of case	Age-adjusted incidence (95% CI)	Incidence rate ratio	No. of cases	Age-adjusted incidence (95% CI)	Incidence rate ratio
Total	81636	19.2 (19.1–19.4)	—	21954	5.1 (5.0–5.2)	—
Histologic subtype						
Squamous cell carcinoma	63943	15.1 (15.0–15.2)	Reference	14360	3.4 (3.3–3.4)	Reference
Adenocarcinoma	1334	0.3 (0.3–0.3)	0.02 (0.02–0.02)	3248	0.8 (0.7–0.8)	0.22 (0.22–0.23)
Other	16359	3.8 (3.8–3.9)	0.26 (0.25–0.26)	4346	1.0 (1.0–1.0)	0.30 (0.29–0.31)
Race						
Whites	72911	20.3 (20.1–20.4)	Reference	17715	4.9 (4.8–5.0)	Reference
Blacks	6379	14.1 (13.7–14.5)	0.70 (0.68–0.71)	3283	7.6 (7.3–7.9)	1.56 (1.50–1.62)
Others	2346	10.9 (10.5–11.4)	0.54 (0.52–0.56)	956	4.3 (4.1–4.6)	0.89 (0.83–0.95)
Histologic type X race						
Squamous cell carcinoma						
Whites	57140	15.9 (15.8–16.1)	Reference	11624	3.2 (3.1–3.3)	Reference
Blacks	5379	11.8 (11.5–12.1)	0.74 (0.72–0.76)	2168	5.1 (4.8–5.3)	1.58 (1.50–1.65)
Others	1424	6.6 (6.2–7.0)	0.41 (0.39–0.44)	568	2.6 (2.4–2.8)	0.82 (0.75–0.89)
Adenocarcinoma						
Whites	1094	0.3 (0.3–0.3)	Reference	2625	0.7 (0.7–0.7)	Reference
Blacks	175	0.4 (0.3–0.5)	1.31 (1.10–1.54)	466	1.0 (0.9–1.1)	1.44 (1.30–1.59)
Others	65	0.3 (0.2–0.4)	1.01 (0.77–1.31)	157	0.7 (0.6–0.8)	0.95 (0.80–1.12)
Other malignancies						
Whites	14677	4.1 (4.0–4.1)	Reference	3466	1.0 (0.9–1.0)	Reference
Blacks	825	1.9 (1.8–2.0)	0.47 (0.43–0.50)	649	1.5 (1.4–1.6)	1.59 (1.46–1.74)
Others	857	4.0 (3.8–4.3)	1.00 (0.93–1.07)	231	1.0 (0.9–1.2)	1.08 (0.94–1.24)

The VUC SCC incidence rate was significantly higher among whites (15.9) than among blacks (11.8) and others (3.5). The VUC ADE incidence rates were similar in whites (0.3), blacks (0.4), and others (0.3). In contrast, the VAC SCC and ADE incidence rates were higher among blacks (5.1 and 1.0, respectively) than among whites (3.2 and 0.7) and others (2.6 and 0.7). The OM incidence rate of VUC was highest among whites (4.1) but of VAC among blacks (1.5) (Table 1).

The incidence rate overall varied regionally and was higher in the Midwest (21.4) and Northeast (21.2) for VUC and the South (5.6) for VAC. Moreover, the incidence rates were lowest in the West for VUC (15.5) and VAC (4.6), respectively. Furthermore, a similar regional difference in the incidence rate was observed within most subgroups stratified by histological type and race (Table 2).

**TABLE 2:** Age-adjusted incidence rates of vulvar and vaginal cancers by histology, race, and region (cohort study, United States, 2001–2018).

Category	Vulvar cancer			Vaginal cancer		
	No. of cases	Age-adjusted incidence (95% CI)	Incidence rate ratio	No. of cases	Age-adjusted incidence (95% CI)	Incidence rate ratio
Total	81636	19.2 (19.1–19.4)	—	21954	5.1 (5.0–5.2)	—
Region						
Northeast	17467	21.2 (20.9–21.6)	Reference	4114	5.0 (4.8–5.1)	Reference
Midwest	20296	21.4 (21.1–21.7)	1.01 (0.99–1.03)	4742	5.0 (4.8–5.1)	1.00 (0.96–1.04)
South	29474	19.0 (18.8–19.2)	0.90 (0.88–0.91)	8790	5.6 (5.5–5.7)	1.12 (1.08–1.17)
West	14399	15.5 (15.3–15.8)	0.73 (0.72–0.75)	4308	4.6 (4.5–4.8)	0.93 (0.89–0.97)
Histologic type X region						
Squamous cell carcinoma						
Northeast	13554	16.5 (16.2–16.8)	Reference	2715	3.3 (3.2–3.4)	Reference
Midwest	16310	17.3 (17.0–17.5)	1.05 (1.02–1.07)	3113	3.3 (3.1–3.4)	0.99 (0.94–1.05)
South	23462	15.2 (15.0–15.3)	0.92 (0.90–0.94)	5761	3.7 (3.6–3.8)	1.12 (1.07–1.17)
West	10617	11.4 (11.2–11.7)	0.69 (0.68–0.71)	2771	3.0 (2.9–3.1)	0.91 (0.86–0.96)
Adenocarcinoma						
Northeast	257	0.3 (0.3–0.4)	Reference	554	0.7 (0.6–0.7)	Reference
Midwest	318	0.3 (0.3–0.4)	1.07 (0.91–1.27)	723	0.8 (0.7–0.8)	1.12 (1.00–1.26)
South	506	0.3 (0.3–0.4)	1.04 (0.89–1.21)	1343	0.8 (0.8–0.9)	1.25 (1.13–1.39)
West	253	0.3 (0.2–0.3)	0.88 (0.74–1.05)	628	0.7 (0.6–0.7)	0.99 (0.88–1.11)
Other malignancies						
Northeast	3656	4.4 (4.3–4.6)	Reference	845	1.0 (1.0–1.1)	Reference
Midwest	3668	3.8 (3.7–4.0)	0.87 (0.83–0.91)	906	1.0 (0.9–1.0)	0.92 (0.84–1.02)
South	5506	3.5 (3.5–3.6)	0.80 (0.77–0.83)	1686	1.1 (1.0–1.1)	1.04 (0.96–1.14)
West	3529	3.8 (3.7–4.0)	0.86 (0.82–0.90)	909	1.0 (0.9–1.0)	0.95 (0.86–1.05)
Race X region						
whites						
Northeast	15808	22.3 (22.0–22.7)	Reference	3344	4.7 (4.6–4.9)	Reference
Midwest	18794	22.1 (21.8–22.4)	0.99 (0.97–1.01)	3980	4.6 (4.5–4.8)	0.98 (0.94–1.03)
South	25402	20.1 (19.8–20.3)	0.90 (0.88–0.92)	6764	5.3 (5.1–5.4)	1.11 (1.07–1.16)
West	12907	16.7 (16.4–17.0)	0.75 (0.73–0.76)	3627	4.7 (4.5–4.8)	0.99 (0.94–1.04)
blacks						
Northeast	1273	15.2 (14.3–16.1)	Reference	621	7.6 (7.0–8.3)	Reference
Midwest	1140	14.4 (13.5–15.3)	0.95 (0.87–1.03)	624	8.4 (7.8–9.1)	1.10 (0.98–1.24)
South	3484	14.1 (13.6–14.6)	0.93 (0.87–0.99)	1791	7.6 (7.2–8.0)	0.99 (0.91–1.09)
West	482	11.6 (10.6–12.7)	0.77 (0.69–0.85)	247	6.1 (5.4–7.0)	0.80 (0.69–0.94)
Others						
Northeast	386	10.8 (9.7–12.0)	Reference	149	4.1 (3.4–4.8)	Reference
Midwest	362	17.2 (15.3–19.2)	1.59 (1.36–1.86)	138	6.4 (5.3–7.6)	1.56 (1.21–2.01)
South	588	13.6 (12.4–14.8)	1.26 (1.09–1.45)	235	5.3 (4.6–6.1)	1.30 (1.04–1.63)
West	1010	8.9 (8.4–9.5)	0.83 (0.73–0.94)	434	3.7 (3.4–4.1)	0.92 (0.75–1.12)

The VUC and VAC incidence rates increased with age and were highest among patients aged 80 years or older overall and for all histological types (Supplementary File).

### Overall Trends in Age-Adjusted Incidence Rates

**FIGURE 1:**
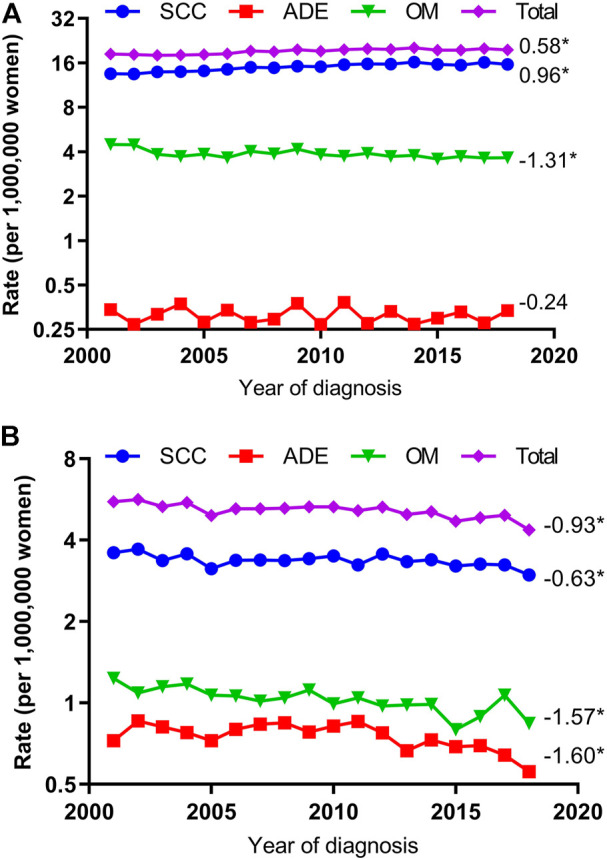
Age-Adjusted Incidence Trends of Vulvar and Vaginal Cancers for Different Histological Types (Cohort Study, United States, 2001–2018). **(A)** vulvar cancer; **(B)** vaginal cancer. All trends are summarized by the average annual percentage change estimated; trends are plotted on a different scale. SCC, squamous cell carcinoma; ADE adenocarcinoma; OM, other malignancies. (*) Significantly different than zero at *p* < 0.05.

Overall, the VUC age-adjusted incidence rate increased from 18.3 per 1,000,000 woman-years in 2001 to 19.6 per 1,000,000 woman-years in 2018 (AAPC, 0.58; 95% CI, 0.24 to 0.91) and VAC age-adjusted incidence rate decreased from 5.6 per 1,000,000 woman-years in 2001 to 4.4 per 1,000,000 woman-years in 2018 (AAPC, −0.93; 95% CI, −1.31 to −0.56, Figure 1).

The VUC SCC incidence rates increased significantly by 0.96 percent yearly (AAPC, 0.96; 95% CI, 0.66–1.25) but VAC SCC incidence rates decreased 0.63 percent annually (AAPC, −0.63; 95% CI, −1.03 to −0.22). The VUC ADE incidence rate was stable (AAPC, −0.24; 95% CI, −1.44 to 0.98) but VAC ADE declined 1.60 percent annually (AAPC, −1.60; 95% CI, −2.80 to −0.39). The VUC OM incidence rate declined 1.31 percent yearly (AAPC, −1.31; 95% CI, −2.58 to −0.02) and VAC OM incidence rate decreased 1.57 percent annually (AAPC, −1.57; 95% CI, −2.24 to −0.89) (Figure 1).

### Trends in Age-Adjusted Incidence Rates by Histologic Type and Race

**FIGURE 2:**
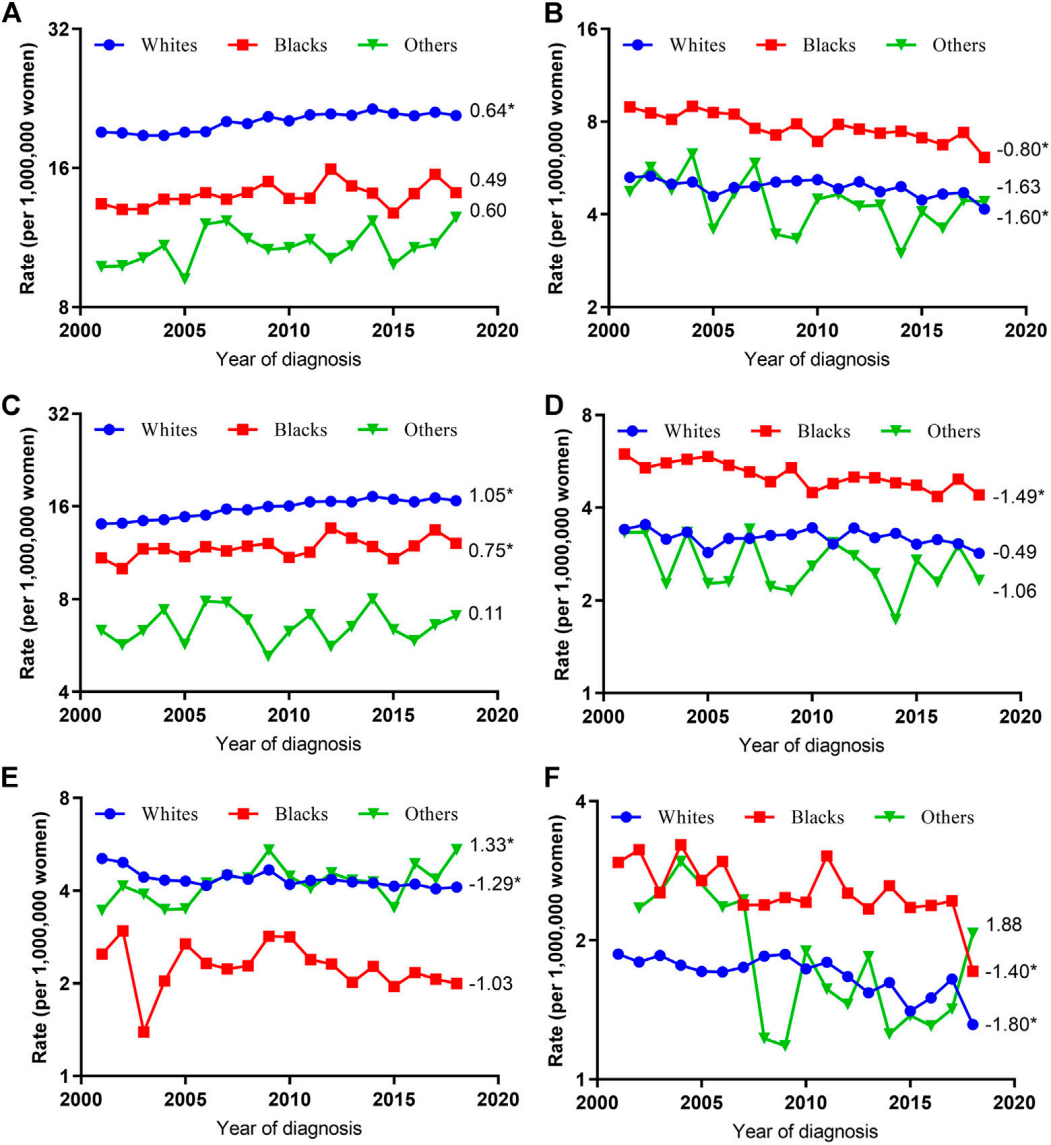
Age-Adjusted Incidence Trends of Vulvar and Vaginal Cancers by Race Overall and by Different Histological Types (Cohort Study, United States, 2001–2018). **(A,C,E)** vulvar cancer; **(B,D,F)** vaginal cancer; **(A,B)** overall and within **(C,D)** SCC and **(E,F)** non-SCC subgroups. All trends are summarized by average annual percentage change estimate; trends are plotted on a different scale. The numbers on the right of the trends curve are average annual percent changes. SCC, Squamous cell carcinoma; non-SCC, adenocarcinoma and other malignancies. (*) Significantly different than zero at *p* < 0.05.

When grouped by race, the VUC SCC incidence rates increased significantly among whites (AAPC, 1.05; 95% CI, 0.78–1.32) and blacks (AAPC, 0.75; 95% CI, 0.10–1.41) but were stable among others (AAPC, 0.11; 95% CI, −1.14–1.37), while VUC non-SCC incidence rates decreased in whites (AAPC, -1.29; 95% CI, −2.34 to −0.23), stabilized in blacks (AAPC, −1.03; 95% CI, −2.54 to 0.50), and increased significantly in others (AAPC, 1.33; 95% CI, 0.11–2.27). As for VAC, SCC and non-SCC incidence rates decreased in all race subgroups, although the decreased incidence rates were only statistically significant among blacks (AAPC, −1.49; 95% CI, −2.01 to −0.98) for SCC and among whites (AAPC, −1.40; 95% CI, −2.01 to −0.78) and blacks (AAPC, −1.80; 95% CI, −2.84 to −0.76) for non-SCC (Figure 2).

### Trends in Age-Adjusted Incidence Rates by Histologic Type and Age

**FIGURE 3:**
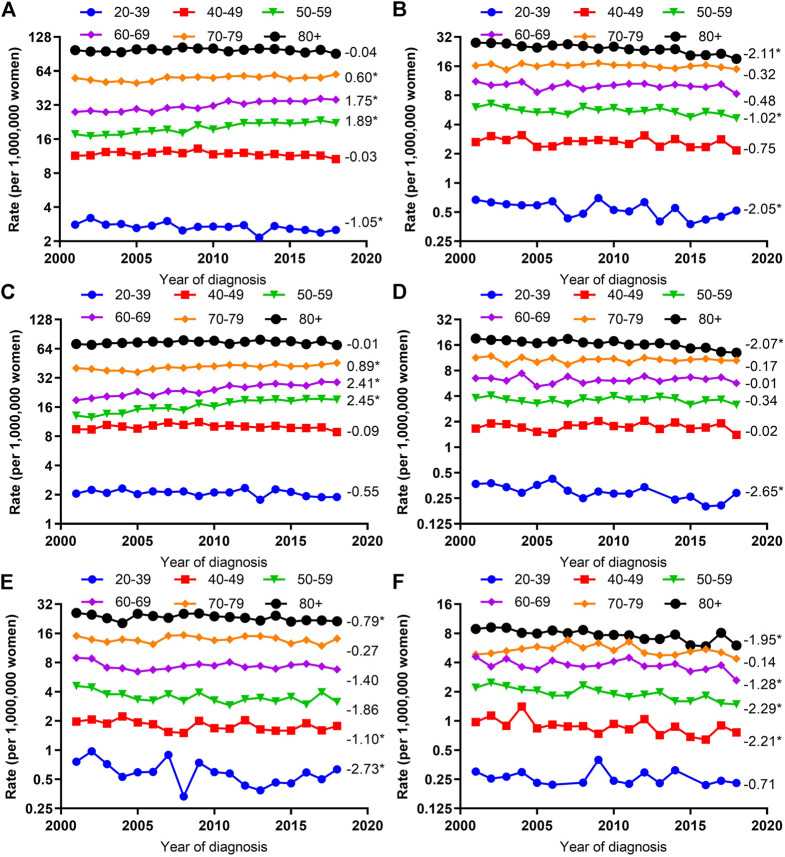
Age-Adjusted Incidence Trends of Vulvar and Vaginal Cancers by Age Overall and by Different Histological Types (Cohort Study, United States, 2001–2018). **(A,C,E)** vulvar cancer; **(B,D,F)** vaginal cancer; **(A,B)** overall and within **(C,D)** SCC and **(E,F)** non-SCC. All trends are summarized by the average annual percentage change estimated; trends are plotted on a different scale. The numbers on the right of the trends curve are average annual percent changes. SCC, squamous cell carcinoma; non-SCC, adenocarcinoma and other malignancies. (*) Significantly different than zero at *p* < 0.05.

After stratifying by age group, significantly increased VUC SCC incidence rate were seen among those aged 50–59 (AAPC, 2.45; 95% CI, 1.64–3.27), 60–69 (AAPC, 2.41; 95% CI, 2.00–2.82), and 70–79 (AAPC, 0.89; 95% CI, 0.58–1.21); however, significantly decreased VUC non-SCC incidence rate were seen among those aged 20–39 (AAPC, -2.73; 95% CI, −4.87 to −0.55), 40–49 (AAPC, 1.10; 95% CI, −2.09 to −0.10), and 80 years or older (AAPC, −0.79; 95% CI, −1.40 to −0.17). On the contrary, the significantly declined VAC SCC incidence rates were observed among those aged 20–39 (AAPC, −2.65; 95% CI, −4.03 to −1.25), 80 years or older (AAPC, −2.07; 95% CI, −2.89 to −1.24), and significantly declined VAC non-SCC incidence rates among those aged 40–49 (AAPC, −2.21; 95% CI, −3.78 to −0.62), 50–59 (AAPC, −2.29; 95% CI, −3.11 to −1.46), 60–69 (AAPC, −1.28; 95% CI, −2.38 to −0.17), and 80 years or older (AAPC, −1.95; 95% CI, −2.77 to −1.12) (Figure 3).

### Trends in Age-Adjusted Incidence Rates by Histological Type and Region

VUC SCC incidence rates increased significantly in the Midwest (AAPC, 1.62; 95% CI, 1.12–2.12), Northeast (AAPC, 1.06; 95% CI, 0.69–1.42), South (AAPC, 0.87; 95% CI, 0.38–1.36), but stabilized in the West (AAPC, 0.27; 95% CI, −0.27–0.81). However, VUC non-SCC incidence rates decreased significantly in the Midwest (AAPC, −1.73; 95% CI, −3.38 to −0.06), South (AAPC,-0.92; 95% CI, −1.66 to −0.18), and West (AAPC, −0.70; 95% CI, −1.37 to −0.02), but not significantly in the Northeast (AAPC, −0.99; 95% CI, −2.45 to 0.49).

**FIGURE 4:**
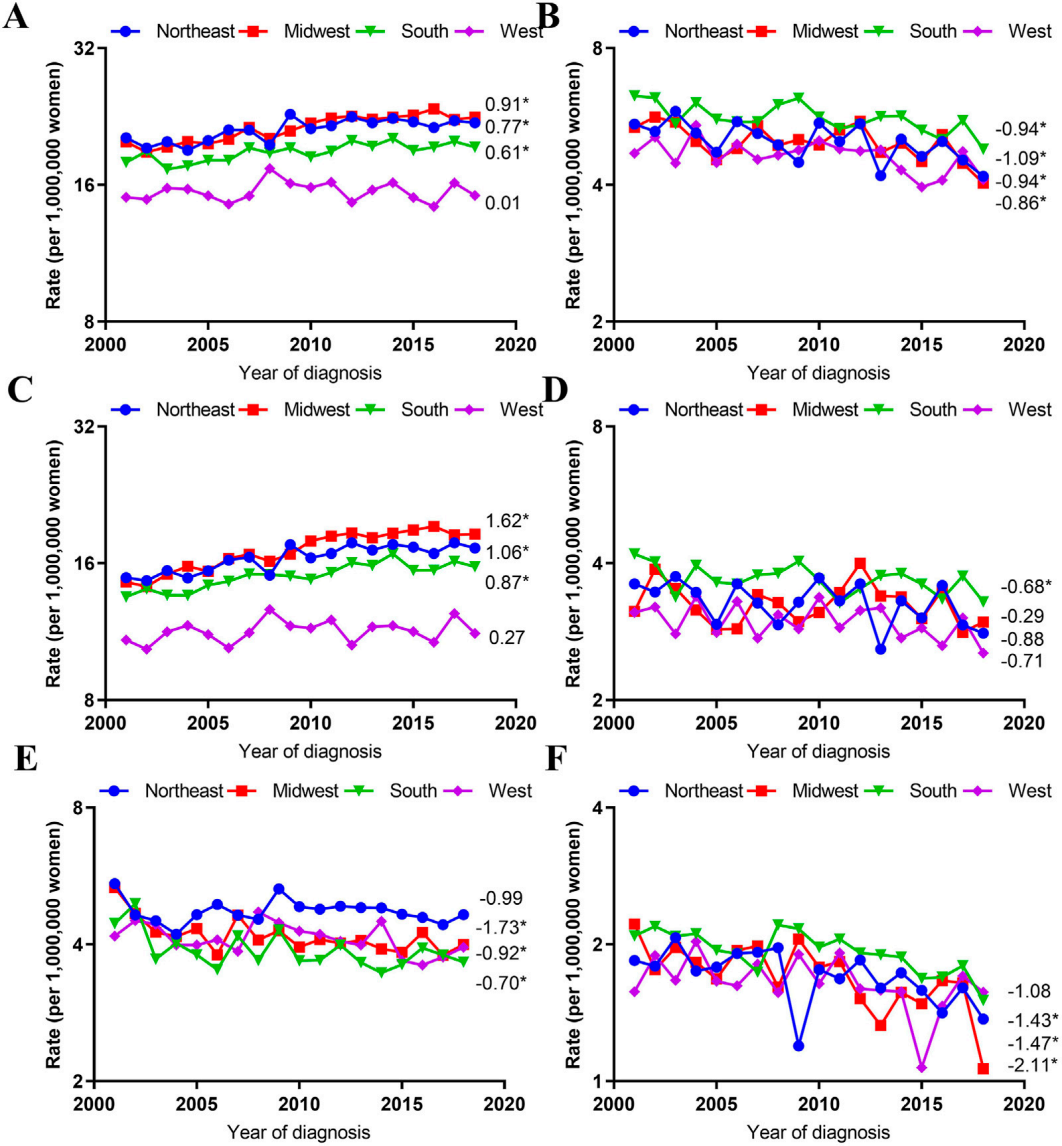
Age-Adjusted Incidence Trends of Vulvar and Vaginal Cancers by Region Overall and by Different Histological Types (Cohort Study, United States, 2001–2018). **(A,C,E)** vulvar cancer; **(B,D,F)** vaginal cancer; **(A,B)** overall and within **(C,D)** SCC and **(E,F)** non-SCC. All trends are summarized by the average annual percentage change estimated; trends are plotted on a different scale. The numbers on the right of the trends curve are average annual percent changes. SCC, Squamous cell carcinoma; non-SCC, adenocarcinoma and other malignancies. (*) Significantly different than zero at *p* < 0.05.

Additionally, VAC SCC incidence rates only declined significantly in the South (AAPC, -0.68; 95% CI, −1.29 to −0.07), but stabilized in other regions. On the contrary, VAC non-SCC incidence rates decreased significantly in Midwest (AAPC, −2.11; 95% CI, −3.23 to −0.98), Northeast (AAPC, −1.47; 95% CI, −2.49 to −0.43), South (AAPC, −1.43; 95% CI, −2.14 to −0.72), but not significantly in the West (AAPC, −1.08; 95% CI, −2.23 to 0.09) (Figure 4).

## Discussion

The critical findings of this study are that VUC and VAC incidence rates for different histology varied significantly by race, age, and region. The VUC SCC incidence rates were increasing, while the VUC non-SCC, VAC SCC, and VAC non-SCC incidence rates were decreasing. Similar incidence rate trends were observed in most stratifications after stratifying by race, age, and region.

As we discovered in this study, the VUC SCC incidence rates were increasing in the United States, similar to a previously published study evaluating the VUC SCC incidence based on the USCS database [5]. The increasing VUC incidence rates were also found in Norway, Germany, and Australia [17–20]. Although based on the incidence rate changes in different histological types and races, this research could probably rule out the probability that the increasing VUC incidence rate was attributable to misclassifying VAC into VUC. It remains unclear why VUC and VAC incidence rates have opposite temporal trends, although they are all Human Papillomavirus (HPV)-related. Unknown risk factors likely contribute to the increasing VUC SCC incidence rate and the decreasing VAC incidence rates, for example, environmental factors or genetic susceptibility, which we could not clarify.

This study found that race was an important factor affecting the incidence rate of VUC and VAC. Blacks had the highest incidence of VAC with any histology type; however, Whites had the highest VUC SCC incidence. One explanation lies in the difference in HPV prevalence. It has been found that black females have a higher high-risk HPV prevalence than Whites [21, 22]. In recent years, the prevention of HPV-related precancers has made significant progress. By 2012, 6 years after the mid of 2006 when HPV vaccination was recommended for females aged 11–26 years old, the HPV-positive cervical precancer incidence decreased in young females aged 14–24 in the United States [23]. From 2008 to 2014, the decreased HPV positive cervical precancer incidence decreased not only in vaccinated females but also in unvaccinated females, but more sharply in vaccinated females [24]. From 2009 to 2011, the percentage of females and males aged 11–18 initiating vaccination increased yearly [25]. The decreased HPV incidence in unvaccinated females might indicate that HPV vaccine introduction had led females to transfer from an unhealthier lifestyle and be more careful to protect themselves from risk factors of HPV, such as an early age for first sexual intercourse, two or more sex partners, and smoking [22]. Another possible explanation might be the unmet healthcare need for black females compared to white females [26]. As for the possible cause of the highest VUC SCC in Whites, there might be unknown factors except for HPV infection or unmet healthcare needs, such as genetic determinants [27].

Our study confirmed the increasing incidence rates in vulvar SCC patients aged 50–59 and 60–69 years in the United States during the past decades, consistent with the findings of the published study [5]. Additionally, we observed an increased VUC SCC incidence among patients aged 70–79 years. Moreover, we found a decreased VAC SCC incidence rate in the 20–39 years and 80 years or older groups. Similarly, the previous study also found decreased VAC SCC incidence rates in women aged younger than 40 and 70 years or older [5]. We also found a decreasing VAC non-SCC incidence rate among patients aged 40–49, 50–59, 60–69, and 80 or older. We confirmed that the oldest patients had the highest incidence rate of VUC and VAC, regardless of the type of cancer, which is possibly contributed to by the accumulation of mutations and exposure-dependent changes in tissue with the increase of age, similar to the pattern in other cancers [28].

To the best of our knowledge, another strength of this study is assessing incidence by United States geographic region. Overall, the VUC incidence rates were higher and increasing more sharply in the Midwest and Northeast, and the VAC incidence rates were highest in the South and decreased in all regions. Furthermore, after stratifying by race, significant regional differences in incidence rates for different histology were observed within most stratifications, underscoring the geographic region’s importance as a factor associated with VUC and VAC incidence rates. Notably, the West region had the lowest and most stable VUC incidence rate and the lowest and decreasing VAC incidence rate. One potential reason for that may be the higher HPV vaccine uptake rate for young female adolescents in the west region of the United States [7, 29]. However, it is worth stating that many years might be spent from vaccination initiation to a future decrease in the incidence rate in specific cancers. Clarifying the reason for the best situation of the West region is of great importance, which may facilitate the improvement of incidence rates of those two cancers in other regions.

Another strength of this study is the detailed incidence rate trends for different histological types within stratifications by race, age, and region. Additionally, the stability of this population-based cohort (USCS) ensures that the same geographic regions are represented over the entire duration of the study. Moreover, the detailed incidence records of newly diagnosed cases and an extended period allow for AAPC calculations and trend observation in this study. Furthermore, a large sample size of patients aged 20 or older, almost complete follow-up data, and high-quality control of the USCS program, make our results representative and generalizable.

Given the racial disparities in VUC and VAC incidence rates, clinicians and policymakers could have a chance to pay more attention to individuals in particular races with higher cancer incidence rates, which will favor making clear the reason for the disparities. The results of this study would encourage policymakers to inspect whether the healthcare delivery needs of patients in different racial groups are fully met or not, which is of great benefit. Ongoing surveillance for the two cancers using high-quality population-based registries is crucial for determining the causes of divergent incidence rates and trends.

Our study was subjected to some limitations. It is a cross-section retrospective study. USCS does not provide any information on the established risk factors for VUC and VAC, such as HPV infection, Human Immunodeficiency Virus infection, smoking, and chronic skin conditions (i.e., lichen sclerosis, dystrophies). Therefore, we could not consider these risk factors when assessing VUC and VAC incidence rates. Moreover, although USCS registries use standardized codes and progress to classify race and ethnicity data, the initial collection of that information is carried out by health care facilities and practitioners, and misclassification in a small proportion of cases is possible.

### Conclusion

Incidence rates of VUC and VAC varied significantly for different histological types and within stratifications by age, race, and region. The incidence rates decreased for VUC and VAC with all histology, except for the increasing VUC SCC incidence. Similar incidence rate patterns were observed in most subgroups stratified by race, age, and region. This study demonstrates the most detailed and comprehensive evaluation of the VUC and VAC incidence rates in the United States. Identifying the disparities in incidence rates could facilitate the exploration of the etiology of those two cancers and satisfaction of health care delivery needs that were not met. This study contributes to the public health and the planning of health services literature.

## Data Availability

The datasets analyzed in this study are available in the SEER database, https://seer.cancer.gov/.
