# Supplementary material for: Trends in the Incidence of Vulvar and Vaginal Cancers With Different Histology by Race, Age, and Region in the United States (2001–2018)
Source: Int J Public Health. 2022 Aug 29;67:1605021. doi: 10.3389/ijph.2022.1605021 (PMC9464823; doi:10.3389/ijph.2022.1605021)
Supplement: Supplementary file 1 [file Table1.DOCX]

**Table. Age-adjusted Incidence Rates of Vulvar and Vaginal Cancers by Histology and Age Group (Cohort Study, United States, 2001-2018)**

| Category | Vulvar cancer | | | Vaginal cancer | | |
| --- | --- | --- | --- | --- | --- | --- |
|  | No. of Cases | Age-adjusted Incidence (95% CI) | Incidence Rate Ratio | No. of Cases | Age-adjusted Incidence (95% CI) | Incidence Rate Ratio |
| Total | 81636 | 19.2(19.1-19.4) | - | 21954 | 5.1(5.0-5.2) | - |
| Age group |  |  |  |  |  |  |
| 20-39 | 3743 | 2.7(2.6-2.8) | Reference | 756 | 0.5(0.5-0.6) | Reference |
| 40-49 | 9324 | 11.9(11.6-12.1) | 4.43(4.27-4.60) | 2080 | 2.6(2.5-2.8) | 4.91(4.52-5.35) |
| 50-59 | 14818 | 20.3(20.0-20.7) | 7.58(7.31-7.86) | 4045 | 5.5(5.3-5.7) | 10.22(9.45-11.07) |
| 60-69 | 16580 | 32.2(31.7-32.7) | 12.02(11.60-12.46) | 5137 | 10.0(9.7-10.3) | 18.53(17.16-20.03) |
| 70-79 | 17503 | 55.5(54.7-56.3) | 20.71(19.99-21.46) | 5091 | 16.1(15.7-16.6) | 29.88(27.67-32.30) |
| 80+ | 19668 | 97.8(96.4-99.2) | 36.49(35.23-37.80) | 4845 | 24.1(23.5-24.8) | 44.76(41.44-48.40) |
| Histologic type X age group | |  |  |  |  |  |
| Squamous cell carcinoma |  |  |  |  |  |  |
| 20-39 | 2885 | 2.1(2.0-2.2) | Reference | 405 | 0.3(0.3-0.3) | Reference |
| 40-49 | 7914 | 10.1(9.9-10.3) | 4.83(4.63-5.04) | 1378 | 1.8(1.7-1.9) | 5.97(5.34-6.68) |
| 50-59 | 12247 | 16.8(16.5-17.1) | 8.06(7.74-8.40) | 2651 | 3.6(3.5-3.7) | 12.26(11.04-13.65) |
| 60-69 | 12790 | 24.8(24.4-25.3) | 11.91(11.44-12.41) | 3223 | 6.3(6.1-6.5) | 21.32(19.22-23.70) |
| 70-79 | 13121 | 41.6(40.9-42.3) | 19.96(19.17-20.79) | 3390 | 10.7(10.4-11.1) | 36.46(32.88-40.53) |
| 80+ | 14986 | 74.5(73.3-75.7) | 35.74(34.34-37.21) | 3313 | 16.5(15.9-17.1) | 56.04(50.53-62.31) |
| Adenocarcinoma |  |  |  |  |  |  |
| 20-39 | 57 | 0.0(0.0-0.1) | Reference | 127 | 0.1(0.1-0.1) | Reference |
| 40-49 | 140 | 0.2(0.2-0.2) | 4.32(3.16-6.00) | 301 | 0.4(0.3-0.4) | 4.28(3.46-5.31) |
| 50-59 | 244 | 0.3(0.3-0.4) | 8.13(6.08-11.06) | 651 | 0.9(0.8-1.0) | 9.92(8.19-12.11) |
| 60-69 | 315 | 0.6(0.5-0.7) | 14.78(11.12-19.97) | 913 | 1.8(1.7-1.9) | 19.88(16.50-24.15) |
| 70-79 | 299 | 0.9(0.8-1.1) | 22.86(17.19-30.94) | 721 | 2.3(2.1-2.5) | 25.52(21.10-31.08) |
| 80+ | 279 | 1.4(1.2-1.6) | 33.61(25.22-45.54) | 535 | 2.7(2.5-2.9) | 29.98(24.66-36.70) |
| Other malignancies |  |  |  |  |  |  |
| 20-39 | 801 | 0.6(0.5-0.6) | Reference | 224 | 0.2(0.1-0.2) | Reference |
| 40-49 | 1270 | 1.6(1.5-1.7) | 2.93(2.68-3.21) | 401 | 0.5(0.5-0.6) | 3.28(2.78-3.89) |
| 50-59 | 2327 | 3.2(3.0-3.3) | 5.73(5.28-6.21) | 743 | 1.0(0.9-1.1) | 6.54(5.62-7.63) |
| 60-69 | 3475 | 6.8(6.6-7.0) | 12.23(11.32-13.22) | 1001 | 1.9(1.8-2.1) | 12.49(10.79-14.51) |
| 70-79 | 4083 | 12.9(12.6-13.4) | 23.37(21.65-25.25) | 980 | 3.1(2.9-3.3) | 19.93(17.22-23.17) |
| 80+ | 4403 | 21.9(21.2-22.5) | 39.51(36.62-42.66) | 997 | 5.0(4.7-5.3) | 31.90(27.56-37.07) |
